# Interstitial Lung Disease Epidemiology in the Past Three Decades: A Narrative Review

**DOI:** 10.3390/jcm13237350

**Published:** 2024-12-02

**Authors:** Francesco Puppo, Roberto G. Carbone

**Affiliations:** Department of Internal Medicine, University of Genoa, 16132 Genoa, Italy; magister1@mail.com

**Keywords:** epidemiology, interstitial lung disease, incidence, prevalence, registries

## Abstract

Current epidemiological data on interstitial lung disease (ILD) are still poor. The principal cause of the discordant data is associated with a heterogeneous group of respiratory diseases that includes a large number, about 200 families, with low frequency, distinct and sometimes unknown etiology, and different progression. In fact, some conditions spontaneously resolve, whereas others, such as IPF and most non-IPF ILDs, progress to respiratory failure and death despite treatment. Furthermore, epidemiological data are limited. The scope of the narrative review is to report ILD incidence and prevalence in registries from different countries in the last three decades. We identified 20 ILD registries (17 prospective and 3 retrospective) from major countries in Europe (n = 10), Asia (n = 7), North America (n = 2), and Oceania (n = 1). Significant discrepancies in ILD and ILD subtype prevalence and incidence among countries are reported in registries. These discrepancies could be determined by different ethnicities and socioeconomic conditions as well as by updates in disease diagnosis and classification. ILD epidemiological registries are progressively ameliorating through better adherence to updated guidelines and classification codes. An accurate and definite diagnosis and compilation of ILD epidemiological registries will be useful for a more precise monitoring of disease progression and treatment. Future research to identify the populations with the highest risk factors, including genetic and molecular studies, and implementation of disease progression scores are needed to improve ILD clinical assessment.

## 1. Introduction

Current epidemiological data on interstitial lung disease (ILD) are still poor. The principal cause of discordant data is certainly associated with a heterogeneous group of respiratory diseases that includes about 200 ILD types [1,2,3] with low frequency, distinct and sometimes unknown etiology, and different progression [4]. Furthermore, available epidemiological data are limited [5].

ILD can be divided into two categories: (1) ILD of unknown etiology and (2) ILD with known cause. Examples of the first category are idiopathic pulmonary fibrosis (IPF), sarcoidosis, hypersensitivity pneumonitis (HP), and undefined (or post-inflammatory) fibrosis, and examples of the second category are ILDs associated with micro-environmental exposure (i.e., fungal spore inhalation), drug use (i.e., amiodarone treatment), occupational causes, connective tissue diseases (CTDs), and previous pneumonias [3]. ILD etiology is often unknown, therefore the search for a causative factor is an important point for ILD clinical diagnosis and classification.

Some conditions spontaneously resolve, whereas others, such as IPF and most non-IPF ILDs, progress to respiratory failure and death despite treatment.

The aim of this narrative review is to report ILD incidence and prevalence in ILD registries from different countries in the last three decades.

## 2. Methods

We identified 20 ILD registries performed in different countries in the last three decades reporting ILD classified by different etiology. Among these registries, 17 were prospective and 3 were retrospective.

ILD incidence, estimated as the rate of new cases over one year, was reported in all registries except the French one. ILD prevalence was not reported in all registries and was calculated by two different methods: (1) prevalence × 100,000 persons in general population and (2) prevalence estimated as proportion of ILD patients in cohorts from multicenter or single-center studies [6]. Data are reported in tables as total number of cases and as percentage in parentheses.

Originally, idiopathic pulmonary fibrosis (IPF) was classified within interstitial idiopathic pneumonias (IIP) corresponding to the International Classification of Diseases Ninth Revision Clinical Modification (ICD-9 CM) 516.3 code. In 2015, ICD-9 CM codes were replaced by ICD-10 CM codes designed to promote international comparability in collection, processing, classification, presentation, and mortality statistics. The ICD-10 CM codes, maintained by the World Health Organization, include greater details, changes in terminology, and expanded concepts for injury, and other related factors, improving accuracy [7].

The 2002 ATS/ERS criteria improved the accuracy of interstitial lung disease classification [8]. ILDs were identified as diffuse parenchymal lung diseases of known and unknown cause. The latter were divided into IIP, granulomatous interstitial lung diseases, and other rare diseases of the interstitial lung. Notably, new diagnostic procedures such as HRCT and updated pathology findings led to the identification of usual interstitial pneumonia (UIP) and made it possible to distinguish IPF/UIP from other IIPs. In 2013, IIP classification was revised according to the ATS/ERS criteria [9], as shown in Table 1.

Prevalence and incidence in ILD subgroups reported by registries from Europe, Asia, North America, and Oceania were analyzed. The Italian registry was included twice because many ILD data from the first registry were unavailable. Lastly, Aosta Registry, a single-region instance, is reported for the long observational period.

Data are shown in tables according to ICD-9, ICD-10, and ATS/ERS classification as reported in each publication.

**Table 1:** Revised idiopathic interstitial pneumonia classification.

Major Idiopathic Interstitial Pneumonia	
Clinical–Radiological Diagnosis	Clinical–Radiological–Pathological Diagnosis
Idiopathic pulmonary fibrosis	Usual interstitial pneumonia
Idiopathic non-specific interstitial pneumonia	Non-specific interstitial pneumonia
Respiratory bronchiolitis—ILD	Respiratory bronchiolitis
Desquamative interstitial pneumonia	Desquamative interstitial pneumonia
Cryptogenic organizing pneumonia	Organizing pneumonia
Acute interstitial pneumonia	Diffuse alveolar damage
Rare Idiopathic Interstitial Pneumonia	
Idiopathic lymphoid interstitial pneumonia	
Idiopathic pleuroparenchymal fibroelastosis	
Unclassifiable idiopathic interstitial pneumonia	

Note: adapted from Travis WD et al. (Ref. [9]).

## 3. Results

**Figure 1:**
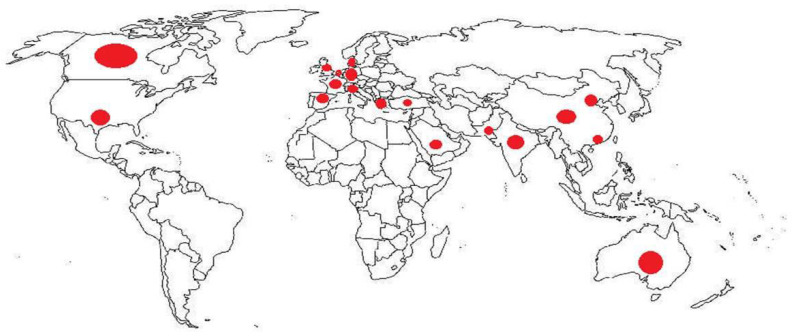
Map showing the most important ILD country registries.

Geographically, all major countries, predominantly from Europe (n = 10) and Asia (n = 7) with respect to North America (n =2) and Oceania (n = 1), were represented in ILD registries (Figure 1).

The first population registry of ILD patients was established in Bernalillo County (NM, USA) in 1988 and included the 1988–1993 period, enrolling 460 ILD patients, with 56% prevalent cases and 44% incident cases. Coultas et al. [1] included IIP associated with IPF in this registry (Table 2).

**Table 2:** Incidence and prevalence of interstitial lung disease (1988–1999 registries).

	New Mexico (USA) ^^^		Flanders ^$^		Germany ^§^	Italy ^#^
Disease	Incidence	Prevalence	Incidence	Prevalence	Incidence	Prevalence
Sarcoidosis	16 (7.9)	30 (11.5)	112 (31)	69 (26)	83 (35)	344 (30)
IIP-IPF	63 (31.2)	58 (22.5)	---	---	---	---
IPF	---	---	62 (17)	50 (19)	76 (32)	417 (37)
BOOP	N/A	N/A	10 (2.3)	9 (3.4)	16 (6.8)	57 (5%)
(C)EP	N/A	N/A	9 (2.5)	7 (2.7)	0	27 (2.3)
CTD-ILD	18 (6.9)	33 (12.8)	27 (7.5)	19 (7.2)	5 (2.1)	N/A
Goodpasture syndrome, GPA, EGPA	N/A	N/A	5 (1.4)	4 (1.5)	2 (0.8)	25 (2.2)
Hypersensitivity pneumonitis	5 (1.5)	N/A	47 (13)	32 (12)	25 (11)	50 (4.3)
Drug	7 (3.5)	5 (11.9)	12 (3.3)	12 (5)	6 (2.6)	21 (1.8)
Eosinophilic granuloma/histiocytosis X	N/A	N/A	13 (3.6)	7 (2.7)	0	73 (7.2)
Pneumoconiosis	21 (10.4)	36 (14.0)	19 (5.0)	18 (6.8)	6 (2.6)	N/A
Post-inflammatory pulmonary fibrosis	20 (9.9)	29 (11.2)	33 (9.1)	27 (10)	12 (5.1)	N/A
Others	54 (26.7)	67 (26.0)	13 (3.6)	10 (3.8)	0	124 (11%)

IIP-IPF: idiopathic interstitial pneumonia–idiopathic pulmonary fibrosis (see Ref. [1]); BOOP: bronchiolitis obliterans organizing pneumonia; (C)EP: (chronic) eosinophilic pneumonia; CTD-ILD: connective tissue disease–interstitial lung disease; GPA: granulomatosis with polyangiitis (Wegener’s); EGPA: eosinophilic granulomatosis with polyangiitis (Churg–Strauss). Registry years: ^ 1988–1993, ^$^ 1992–1996, ^§^ 1995, ^#^ 1997–1999. Data from the registries published after the year 2000 are then reported.

In 2001 Thomeer et al. [10] reported three registries that included ILD cases in Flanders (Belgium), Germany, and Italy in the 1992–1999 period. The incidence of CTD-ILD and undefined fibrosis was higher in Flanders than in Germany. In Flanders, there was a slight predominance of male patients (especially in ILD due to occupational exposure and/or hypersensitivity pneumonitis), whereas there was a female predominance in Germany. Drug-induced ILDs were found in oldest patients in Flanders, particularly those due to amiodarone and nitrofurantoin. A low prevalence of hypersensitivity lung diseases was observed in Italy and a lower percentage of IPF was present in Belgium than in Germany and Italy (Table 2).

Data from European registries are summarized in Table 3.

Total ILD incidence in Spain and Greece ranged between 3 and 7 × 100,000 inhabitants. Moreover, IPF incidence was lower in Greece whereas sarcoidosis incidence was lower in Spain [11,12,13]. The Turkey registry showed that sarcoidosis incident cases were the most common within ILD patients (771 cases). Data from the Danish population registry report that the total ILD incidence was 23 × 100,000 inhabitants, with a higher incidence among males (around 29%) [12,13]. Data from the French registry indicated a higher prevalence of sarcoidosis (361 cases) and CTD-ILD (145 cases) with respect to other ILD [14].

IPF rate reported in the United Kingdom was approximately 5 × 100,000 [15]. This finding is close to the rate calculated in the USA considering a more restricted classification criterion of the disease, while a significantly higher incidence occurs considering broader criteria. The incidence of IPF increased over the years in the United Kingdom (from 3% in 1991–1995 to 7% in 2000–2003). Of note, whereas the United Kingdom study indicated that the incidence of IPF was more than doubled during the observation period [15], the population-based data from the USA (Olmsted County) and Denmark [6,16] showed a declining incidence, particularly during the latter years of the study.

The registry for diffuse infiltrative lung disease named RIPID (Registro Italiano Pneumopatie Infiltrative Diffuse) was created in Italy in 1998 with the scope of obtaining a complete national ILD database. During the 1998–2005 period, a total of 3152 ILD patients were registered [17]. However, the incidence could not be estimated because the size of the population covered by the participating centers was not exactly estimable, except for Bolzano province in the first instance and subsequently Aosta region in the period from 2005 to 2018.

Data from Asia, Canada, and Australia are reported in Table 4. Recently, the largest retrospective epidemiology study in Asia was performed by Ye et al. [5] in Hong Kong, including 924 ILD patients observed over a 16-year period from January 2005 to December 2020. Global ILD prevalence increased from 23.1 × 100,000 in 2005 to 32.6 × 100,000 in 2020 while the incidence decreased from 5.97 × 100,000 to 3.83 × 100,000 inhabitants in the same period. This finding could be related to a better quality of diagnostic procedures in association with adherence to updated classification guidelines. The study showed higher accuracy with respect to other Asia registries because ILD case identification was validated according to updated guidelines by chest imaging, physical medical notes, and clinical examinations, and was made by specialists in respiratory medicine. ILD was divided into two categories: (1) post-inflammatory pulmonary fibrosis and (2) idiopathic interstitial pneumonia coded 515 and 516.3, respectively, in accordance with ICD-9-CM and ICD-10-CM diagnostic codes [5,7].

Interestingly, the study compared data from Asia with those from other countries and reported that ILD prevalence ranged from 6.27 × 100,000 subjects in Belgium to 97.9 × 100,000 subjects in Paris and that ILD incidence ranged from 1 to 32 × 100,000 person-years in Europe, the United States (New Mexico registry), the Middle East, and Asia. The prevalence of ILD in Hong Kong was like in Greece but lower than in Paris and in the United States and approximately four-fold higher than in Belgium. The overall ILD incidence in Hong Kong was 5.21 × 100,000 subjects, which was comparable to Greece, Spain, and Central Denmark, but was lower than in India, the United States, Turkey, and Paris.

A Registry reporting ILD incidence and prevalence in India (tri-city region) was published in 2022 [18]. Incident cases were 159, 887, 70, and 58 for sarcoidosis, IPF, CTD-ILD, and HP, respectively. In the same study, the national population incident ILD cases were calculated, indicating a strong incidence of HP (513 cases) [18]. The elevated rate of HP in India could be attributable to two opposite realties, namely, the presence of high-tech areas in contrast with rural agricultural regions.

Data from Chinese registries indicated that IPF was the most frequent subtype, followed by CTD-ILD [14]. Analogous findings were present in the Australian registry [14,19]. By contrast, CTD-ILD was the highest ILD subtype in the Canadian registry [14,20].

In 2021, Olson et al. [21] published a study on the prevalence and incidence of chronic fibrosing interstitial lung diseases the United States. Although the study could not be considered a registry, it is the first one aimed to estimate ILD prevalence and incidence in the United States, evaluating a total of 37,565,644 patients aged at least 18 years identified from 1 October 2012 to 30 September 2015 with at least 365 days’ continuous enrollment. The estimated age- and sex-adjusted prevalence per 100,000 persons of fibrosing ILD (IPF) was 117.8 (95% CI, 116–119). Notably, the study estimated that incidence per 100,000 patient-years of fibrosing ILD and chronic fibrosing ILD with a progressive phenotype was 51.5 and 32.5, respectively. ILD identification without specific diagnosis codes were defined idiopathic non-specific interstitial pneumonia (INSIP) or unclassifiable idiopathic interstitial pneumonia (unclassifiable IIP) based on ICD-9 CM. After an accurate screening the Authors concluded that ILD/IPF incident cases were 57% out of a total of 15,518 cases. The remaining cases were INSIP, unclassifiable IIP and CTD-ILD, occupational ILD, and hypersensitivity pneumonitis.

### A Single-Region Experience: Aosta Registry

**Figure 2:**
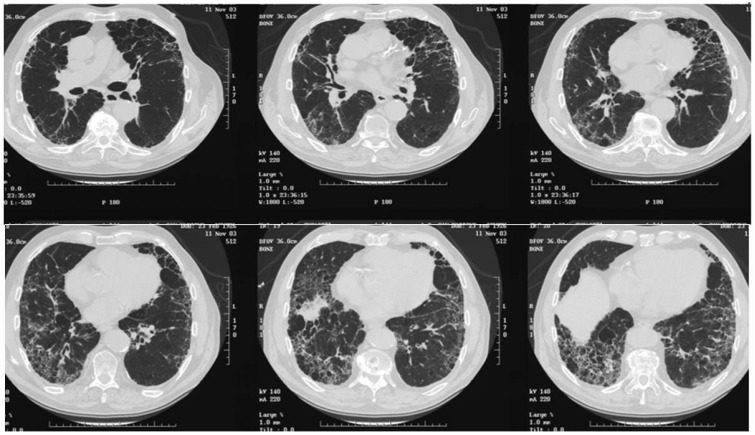
Series of chest HRCT images showing IPF characterized by reticular pattern and honeycombing associated with pulmonary emphysema.

**Figure 3:**
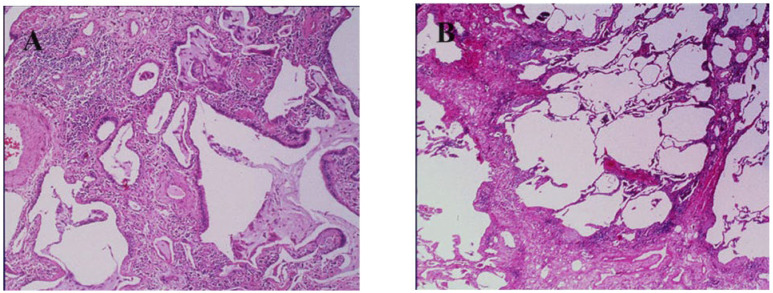
Pathological findings of pulmonary lesions in usual interstitial pneumonia showing patchy involvement of the pulmonary parenchima (**A**) and interalveolar septa (**B**) by a fibrotic process.

In 1995–2004 period 128 ILD patients were enrolled in Aosta region, Italy (126,000 inhabitants) (Table 5). According to the American Thoracic Society Criteria recommendation [22], the diagnosis was made on clinical, radiological, and histological data: 59 patients were diagnosed with IPF/UIP, 19 with NSIP, and the remaining 50 patients with sarcoidosis (n. 24), GPA (n. 16), and extrinsic allergic alveolitis (n. 10). All ILD diagnoses were confirmed by lung biopsy [23]. Interestingly, the registry was updated in 2018, including data from 2005 to 2018, and showed an increase in ILD incidence, also including ILD associated with GPA (Wegener’s) and hypersensitivity alveolitis (data not published). These data are at odds with other registries, in which the most frequent diseases were IPF and sarcoidosis. ILD incidence was 7 × 100,000 inhabitants. The accuracy of ILD diagnosis and registration was facilitated because patients were part of a region with a numerically reduced population of residents, they were referred to a single hospital center, and update courses on pulmonary fibrosis for general practitioners were implemented in close collaboration with hospital specialists. IPF high-resolution computed tomography (HRCT) scan and histopathological features are reported here (Figure 2 and Figure 3A,B).

## 4. Discussion

Chronic respiratory diseases (CRDs) include many pathological conditions like chronic obstructive pulmonary disease (COPD), asthma, pneumoconiosis, interstitial lung disease (ILD), and pulmonary sarcoidosis. In 2019, CRDs were the third cause of death with 4 million deaths and a prevalence of 454.6 million cases around the world, associated with substantial cost. Total CRD prevalence increased from 28.5% to 39.8% in the 1990–2019 period. Globally, the total number of deaths, incidence, and prevalence of CRD rose, whereas age-standardized rates (ASR) declined in both sexes over the past three decades. Nevertheless, this trend was variable among different geographical locations and sexes [23].

Among obstructive respiratory diseases, COPD and asthma have been the main contributors to the global prevalence and incidence of CRD. COPD, with about 212 million prevalent cases, was the first cause of death, accounting for 3.3 million deaths. Asthma accounted for 21.6 million disability-adjusted life years (DALYs) globally in 2019 with 262.4 million prevalent cases and 37.0 million new cases. All measures were closely comparable between the sexes. Asthma constituted the majority of DALYs in the under 35 age group in 2019 worldwide. The age standardized rate of incidence, prevalence, deaths, and DALYs have significantly decreased from 1990 to 2019 by 13.1%, 24.1%, 51.3%, and 42.5%, respectively [23].

Among restrictive respiratory diseases, the age-standardized rate of ILD incidence and prevalence increased from 1990 to 2019 by 9.4% and 14%, respectively. In this period, the ILD age-standardized rate of DALYs and death were lower in women. ILD and sarcoidosis were responsible for 3.8 million DALYs globally in 2019, with 4.7 million prevalent cases and 23.2 million incident cases [21]. Furthermore, in 2019, Asia–Pacific and North America followed by South Asia had the highest age standardized rate of DALYs of incidence and prevalence. In this scenario, IPF is usually considered one major ILD subgroup due to its high prevalence and incidence and poor prognosis [24].

Globally, silicosis, asbestosis, coal workers, and other pneumoconiosis were estimated to account for 0.9 million disability-adjusted life years and 3.1 million prevalent cases in 2019. Pneumoconiosis prevalence remained comparable from 1990 to 2019, but silicosis, asbestosis, and coal worker death and incidence decreased by 44.4%, 53.3%, and 13.7%, respectively, in the same period. Pneumoconiosis accounted for 90% of occupational disease in China. ILD and pulmonary sarcoidosis deaths remained stable whereas their incidence and prevalence increased [25].

More recently, several studies investigating the prevalence and incidence of non-IPF-ILD, especially autoimmune ILD, have been published. A recent review reported that the prevalence of ILD in myositis ranged from 23% to 50% in America and Asia, respectively [26]. Sambataro et al. [27] reported that about 20% of Sjögren syndrome cases developed ILD. The prevalence of ILD in systemic sclerosis (SSc) ranges from 2.3 to 19 per 100,000 persons in Canada and in the USA, respectively, and is 2% in rheumatoid arthritis. Additional studies evaluated the incidence of drug-induced ILD [6].

In the past three decades, a notable decrease the in age-standardized rate of DALYs was observed for all CRD risk factors except for ambient particulate matter (PM) pollution, high body mass index, and occupational asbestos exposure in both sexes. Smoking and ambient PM represent major CRD risk associated with recent climate change, including increased temperature. A targeted campaign against tobacco in China named the Healthy China 2020 strategy, with a scope to reduce smoking prevalence to 20%, can explain the significant reduction of smoking risk in this country.

ILD predicts severity and higher mortality in COVID-19 patients. ILD onset can reduce the outcome of COVID-19 patients and can cause fibrotic-like changes in both lungs, also after 6-12 months. Approximately 25% of survivors of acute respiratory distress syndrome (ARDS) demonstrate a restrictive pattern of lung function at 180 days, and these patients also demonstrated fibrotic changes (reticular infiltrates) on CT imaging. As post-ARDS pulmonary fibrosis is a well-established complication, its potential development as a long-term outcome of COVID-19 is a major concern. Unlike IPF or other progressive ILDs, fibrosis resulting from ARDS tends to be more stable [28].

Environmental pollution, occupational risks, and smoking are principal CRD risk factors with various geographical, culture, age, and sex distributions. Implementation of registries will improve the knowledge on CRD epidemiology and trends and help policymakers in developing targeted interventions to reduce morbidity and mortality.

The registries published in the last three decades show significant discrepancies in prevalence and incidence of ILD and subtypes among different countries. These discrepancies could be determined by different ethnicities and socioeconomic conditions among populations.

For instance, Guo et al. [29] reported that ILD incidence ranged from 4.6 to 31.5 per 100,000 persons in Europe and North America and a recent study indicated that the global ILD incidence in the past 10 years has risen from 207.2 in 1990 to 313.2 cases per 100,000 cases in 2019 [30].

Moreover, the various updates in ILD diagnosis and classification made over the last thirty years can be cause of instrumental limitation for the diagnosis obtaining misdiagnosis and/or lack of inclusion of possible ILD cases in their respective national registry. ILD epidemiological registries are progressively ameliorating through better adherence to updated guidelines and classification codes in disease diagnosis. Variations in prevalence estimates may be attributable to differences between countries and within different healthcare settings in the same country. Notably, most data derive from Hospital files in which only partial data are reported, while ILD population registries are present only in a few cases. Furthermore, selection bias may influence ILD prevalence and incidence in different countries, as data are mainly registered in internal medicine or pneumology units and do not refer to the entire population. Moreover, either prevalence or incidence data or a mixture of both are reported, leading to poor statistical significance. Finally, in most countries, especially in Asia, diagnosis was made only on clinical and radiological findings without histopathological confirmation. Therefore, there is the need for standardization of diagnostic classification criteria and for clear diagnostic guidelines for non-IPF and IPF ILD [6].

Lastly, Carbone et al. [31] demonstrated in 2007 that two pathological findings are detectable in non-specific interstitial pneumonia (NSIP): i.e., cellular NSIP and fibrotic NSIP with a different prognosis, in favor of the first subgroup. The important new “outcome subgroup” to emerge was the entity of fibrotic NSIP. These patients made up 20–35% of patients previously diagnosed as IPF or cryptogenic fibrosing alveolitis (CFA)/idiopathic pulmonary fibrosis (IPF) [32,33]. The detection of a further subset of NSIP lead to a more precise classification of ILD patients but at the same time made registry compilation more complex. 

A limitation of the published studies is a lack of national ILD registries in very large and densely populated countries such as the USA, China, and India. Additionally, several registries do not report pneumoconiosis and unclassifiable fibrosis subsets. Moreover, in some registries, diagnosis was made only on clinical and radiological findings without histopathological confirmation in misdiagnosed cases. Lastly, registries published in different periods are not fully comparable, as they are based on different ILD classifications.

Accurate diagnosis made by multidisciplinary panels and coding practices according to continuously updated guidelines will lead to more precise ILD classification. A definite compilation of ILD epidemiological registries will be useful for better monitoring disease progression and treatment, especially with anti-fibrotic drugs and lung transplantation.

## 5. Conclusions

The present review focuses on regional differences in ILD prevalence and incidence reported in published registries. These discrepancies might be in part attributable to racial genetic differences such as those observed in IPF patients regarding the MUC5B mutation. Additional genetic risk variants outside the MUC5B region, when identified, could better predict ILD progression [34,35].

Furthermore, although several scoring systems (CRP, CPI, and GAP) have been proposed to compare all major variables in IPF patients [36,37,38,39], a comprehensive score including all variables and a standard definition of disease progression is currently lacking.

Future policies, especially in low-income countries, including prevention to identify the populations with the highest risk factors, specialized respiratory care facilities, and enhanced access to diagnostic tools, as well as treatments, are required to reduce ILD burden. Furthermore, implementation of global strategies to reduce tobacco use, air pollution, and occupational hazards are key steps in achieving ILD reduction.

## Figures and Tables

**Table 3:** Incidence and prevalence of interstitial lung disease in Europe.

	Greece *		Spain ^$^	Turkey ^§^	France ^#^	Italy ^&^	Denmark ^^^
Disease	Incidence	Prevalence	Incidence	Incidence	Prevalence	Incidence	Incidence
Sarcoidosis	60 (23.2)	330 (34.1)	76 (14.9)	771 (34.3)	361 (42.6)	1063 (33.7)	N/A
IPF/UIP	52 (20.1)	189 (19.5)	197 (38.6)	408 (18.2)	98 (11.5)	864 (27.4)	121 (28.1)
CTD-ILD	30 (11.6)	120 (12.4)	51 (10.0)	201 (9.0)	145 (17.1)	N/A	54 (12.5)
HP	7 (2.7)	25 (2.6)	34 (6.6)	82 (3.7)	28 (3.3)	93 (2.9)	32 (7.4)
Drug	4 (1.5)	17 (1.8)	17 (3.3)	35 (1.6)	31 (3.7)	39 (1.2)	20 (4.6)
Pneumoconiosis	8 (3.1)	20 (2.0)	N/A	241 (10.7)	42 (5.0)	N/A	N/A
Fibrosis unclassifiable	40 (15.4)	82 (8.5)	26 (5.1)	99 (4.4)	66 (7.8)	N/A	62 (14.4)
Others	58 (22.4)	184 (19.0)	110 (21.5)	408 (18.2)	77 (9.1)	N/A	142 (32.9)

IPF/UIP: idiopathic pulmonary fibrosis/usual interstitial pneumonia; CTD-ILD: connective tissue disease–interstitial lung disease; HP: hypersensitivity pneumonitis. Registries years: * 2004; ^$^ 2000–2001; ^§^ 2007–2009; ^#^ Paris area 2012; ^&^ 1998–2005; ^ 2001–2005.

**Table 4:** Incidence and prevalence of interstitial lung disease in Asia, Canada, and Australia.

	India ^$^		India ^$$^		Pakistan ^§^	China *	China **	Hong Kong ^&^	Saudi Arabia ^^^	Canada ^+^	Australia ^#^
Disease	Incidence	Prevalence	Incidence	Prevalence	Incidence	Incidence	Incidence	Incidence	Incidence	Incidence	Incidence
Sarcoidosis	217 (38.3)	339 (42.2)	85 (7.8)	38 (9.5)	11 (4.3)	123 (6.3)	147 (5.6)	188 (4)	67 (20)	41 (3.2)	44 (6.2)
IPF/UIP	130 (23.0)	170 (21.2)	148 (13.7)	37 (9.3)	95 (37.5)	395 (20.3)	692 (26.5)	3746 (79.8)	77 (23.3)	317 (24.7)	240 (34.0)
CTD-ILD	77 (13.6)	102 (12.7)	151 (13.9)	80 (20.1)	23 (9.1)	356 (18.3)	631 (24.1)	93 (2.0)	115 (34.8)	428 (33.3)	125 (17.7)
HP	69 (12.2)	86 (10.7)	513 (47.3)	190 (47.6)	31 (12.3)	59 (3.0)	62 (2.4)	81 (1.7)	21 (6.3)	97 (7.5)	66 (9.4)
Drug	5 (0.9)	6 (0.7)	N/A	N/A	N/A	13 (0.7)	28 (1.1)	31 (3.7)	4 (1.2)	N/A	7 (1.0)
Pneumoconiosis	6 (1.1)	7 (0.9)	N/A	N/A	3 (1.2)	13 (0.7)	58 (2.2)	N/A	N/A	N/A	11 (1.6)
Fibrosis unclassifiable	N/A	7 (0.9)	N/A	N/A	4 (1.6)	285 (14.7)	344 (13.2)	N/A	6 (1.8)	286 (22.3)	51 (7.2)
Others	62 (11.0)	86 (10.7)	187 (17.3)	54 (13.5)	86 (34.0)	701 (36.0)	653 (25.0)	242 (4.9)	40 (12.1)	116 (9.0)	161 (22.8)

IPF/UIP: idiopathic pulmonary fibrosis/usual interstitial pneumonia; CTD-ILD: connective tissue disease–interstitial lung disease; HP: hypersensitivity pneumonitis. Registry years: ^$^ 2015–2017; ^$$^ 2015–2020; ^§^ 2016–2018; * 2012–2017; ** 2000–2012; ^&^ 2005–2020; ^ 2008–2011; ^+^ 2016–2017, ^#^ 2016–2019.

**Table 5:** Interstitial lung disease registry in Aosta region, Italy (2005–2018).

	Number *	Gender		Age Median (Range)
		Male (%)	Female (%)	
Disease				
NSIP	33 (20.6)	19 (57.6)	14 (42.4)	51 (28–75)
IPF/UIP	73 (45.6)	54 (61.6)	28 (38.4)	64 (27–79)
Sarcoidosis	38 (23.7)	24 (63.2)	14 (36.8)	42 (30–58)
EGPA	16 (10.0)	10 (62.5)	6 (37.5)	64 (48–78)

* Incidence: 7 × 100,000 inhabitants; NSIP: non-specific interstitial pneumonia; IPF/UIP: idiopathic pulmonary fibrosis/usual interstitial pneumonia; EGPA: eosinophilic granulomatosis with polyangiitis (Churg–Strauss).
